# Supplementary material for: Autistic children sample costly information with increased variability due to inflexible updating
Source: Commun Psychol. 2026 Mar 20;4:80. doi: 10.1038/s44271-026-00439-2 (PMC13168704; doi:10.1038/s44271-026-00439-2)
Supplement: Supplementary file 2 — Supplementary Information [file 44271_2026_439_MOESM2_ESM.pdf]

Autistic children sample costly information with increased variability due to inflexible updating

Haoyang Lu[1,2], Hang Zhang[1,3,4,5*], Li Yi[1,3,5*]

[1] School of Psychological and Cognitive Sciences and Beijing Key Laboratory of Behavior and Mental Health, Peking University, Beijing, China; [2] Applied Computational Psychiatry Lab, Max Planck UCL Centre for Computational Psychiatry and Ageing Research, Queen Square Institute of Neurology and Mental Health Neuroscience Department, Division of Psychiatry, UCL, London, United Kingdom; [3] PKU-IDG/ McGovern Institute for Brain Research, Peking University, Beijing, China; [4] Peking-Tsinghua Center for Life Sciences, Peking University, Beijing, China; [5] Key Laboratory of Machine Perception (Ministry of Education), Peking University, Beijing, China

*Correspondence to Hang Zhang (hang.zhang@pku.edu.cn) and Li Yi (yilipku@pku.edu.cn); these authors jointly supervised this work.

Supplementary Note 1: Autistic children differed from neurotypical children in sampling strategy rather than correct judgment

For gained credits in each trial, the linear mixed model found significant main effects of Group (LMM1; $F(1, 70.20) = 10.61$, $p = .002$, $\eta_p^2 = .13$, 95% CI [.02, .28]), Cost ($F(2, 74.33) = 105.87$, $p < .001$, $\eta_p^2 = .74$, 95% CI [.64, .81]), and Evidence ($F(1, 90.27) = 365.42$, $p < .001$, $\eta_p^2 = .80$, 95% CI [.73, .85]), and significant interactions between Group and Cost ($F(2, 74.33) = 6.04$, $p = .004$, $\eta_p^2 = .14$, 95% CI [.02, .28]), and between Group and Evidence ($F(1, 90.27) = 13.02$, $p = .001$, $\eta_p^2 = .13$, 95% CI [.03, .26]). When we examined different conditions separately, we found that autistic children had won significantly fewer credits in the low-cost condition ($M_{ASD} = 71.8$, $M_{NT} = 76.9$, $t(72.2) = –2.52$, $p = .04$, $d = -0.15$, 95% CI [-0.30, -0.005]), the high-cost condition ($M_{ASD} = 54.0$, $M_{NT} = 64.7$, $t(72.6) = –4.10$, $p < .001$, $d = -0.32$, 95% CI [-0.51, -0.13]), and in the high-evidence condition ($M_{ASD} = 77.4$, $M_{NT} = 85.7$, $t(71.3) = –4.99$, $p < .001$, $d = -0.25$, 95% CI [-0.37, -0.14]).

An analysis of the proportion of correct judgment allows us to exclude the possibility that autistic children made more incorrect judgment. There was no significant difference between the two groups in the proportion of correct judgment (LMM2; $\chi^2(1) = 1.70$, $p = .20$). Though a significant interaction effect was found between the group and the evidence conditions ($\chi^2(1) = 7.52$, $p = .006$), post hoc comparisons did not support significant group differences under any of the two evidence conditions (low-evidence condition: $M_{ASD} = 73.1\%$, $M_{NT} = 71.6\%$, $OR = 1.08$, 95% CI: [0.86, 1.36], $p = .70$; high-evidence condition: $M_{ASD} = 91.4\%$, $M_{NT} = 94.4\%$, $OR = 0.64$, 95% CI: [0.40, 1.04], $p = .08$). When comparing two groups in terms of the gained credits in those correct trials, we found that autistic children won fewer credits particularly in those costly trials (interaction between groups and cost conditions: $F(2, 74.96) = 5.27$, $p = .007$, $\eta_p^2 = .12$, 95% CI [.01, .26]; low-cost condition: $t(71.1) = -3.05$, $p = .009$, $d = -0.54$, 95% CI [-0.96, -0.11]; high-cost condition: $t(71.1) = -3.02$, $p = .010$, $d = -1.63$, 95% CI [-2.93, -0.33]).

Supplementary Note 2: Model parameters correlations with sampling bias and variation

Among the model parameters that predicted sampling efficiency, $\beta_{|CumuInfo|}$ was negatively correlated with sampling bias (r_S = -.60, 95% CI [-.73, -.42], p = .001) and variation (r_S = -.39, 95% CI [-.57, -.17], p = .002). $\beta_{CumuCost}$ was only negatively correlated with the sampling variation (r_S = -.32, 95% CI [-.52, -.09], p = .009) but not with sampling bias (r_S = -.21, 95% CI [-.43, -.03], p = .075). $\beta_{LastDraw}$ showed a significant positive correlation with sampling bias (r_S = .36, 95% CI [.13, .55], p = .003) and sampling variation (r_S = .46, 95% CI [.25, .63], $p < .001$). Consistent with the group differences in the parameter and the behavioral measures, α_{Decay} also showed significant negative correlations with sampling bias (r_S = -.30, 95% CI [-.50, -.06], p = .013) and sampling variation (r_S = -.36, 95% CI [-.55, -.14], p = .003), which were also consistent with the group differences in α_{Decay} and these measures of sampling behavior.

Supplementary Note 3: Exploratory dimensional analyses

To explore how dimensional autistic traits influence children's sampling behaviors, we conducted similar behavioral and modeling analyses but replaced the group effect term with the AQ-Child score.

Within a subsample of 51 children who had AQ-Child scores, we first found that children with higher AQ scores tended to have lower sampling efficiency specifically in the high-cost condition (AQ x Cost interaction: F(2, 49.01) = 3.57, p = .036, η_p^2 = .13, 95% CI [.00, .30]; $\beta_{AQ|High\text{-}cost}$ = -0.043, t(49) = -2.53, p = .015). We then examined which domain of autistic traits was most strongly associated with the observed effect, and the results showed that, when holding other AQ subscale scores constant, only Social Skills scores significantly predicted sampling efficiency. The significant AQ-Social Skills x Cost interaction again suggested that children with more social discomfort had higher sampling efficiency when there was no sampling cost, while their sampling performance worsened substantially when sampling incurred higher costs (Social Skills x Cost interaction: F(2, 44.99) = 5.18, p = .009, η_p^2 = .19, 95% CI [.01, .37]).

We then further investigated sampling bias and variation. Consistent with the group-based analysis, we found that children with higher total AQ scores or Social Skills scores sampled more in general (β_{AQ} = 1.17, t(48.9) = 2.44, p = .018; $\beta_{AQ\text{-Social Skills}}$ = 2.14, t(45) = 2.58, p = .013). Particularly, children having higher Social Skills scores tended to sample more when evidence level was high and cost level was low (Social Skills x Cost x Ratio interaction: F(2, 45.01) = 3.60, p = .035, η_p^2 = .14, 95% CI [.00, .32]). For sampling variation, we did not find that AQ total score significantly predicted it; however, there was a significant main effect of Social Skills scores, such that children with more social discomfort had greater between-trial sampling variability ($\beta_{AQ\text{-Social Skills}}$ = 0.59, t(45) = 2.34, p = .023).

Correlational analyses between individual-level parameter estimates and AQ total scores showed patterns directionally consistent with group differences, but most did not survive multiple comparison correction (α_{Decay}: r_S = -.29, 95% CI [-.53, -.01], p = .036, FDR p = .16). Considering that the Social Skills subscale emerged as a uniquely strong predictor for sampling behaviors, we examined the parameter correlations with the Social Skills subscale specifically. We found that only the evidence accumulation decay parameter α_{Decay}—which also showed significant group differences—remained significant after FDR correction (r_S = -.38, 95% CI [-.60, -.11], p = .006, FDR p = .031). Other parameters showed correlations in expected directions but did not reach corrected significance thresholds (β_0: r_S = -.32, 95% CI [-.55, -.04], p = .021, FDR p = .054; $\beta_{L\text{-}0}$: r_S = .32, 95% CI [.04, .55], p = .022, FDR p = .054; $\beta_{|CumuInfo|}$: r_S = -.32, 95% CI [-.55, -.03], p = .024, FDR p = .054; $\beta_{LastDraw}$: r_S = .30, 95% CI [.02, .54], p = .030, FDR p = .055).

Supplementary Methods 1: Participant exclusion

We collected data from 38 autistic children and 47 neurotypical children in total. Five autistic children did not understand the instructions to pass the practice trials, and thus they did not take test trials. Because of emotional distress, one autistic child quit the experiment after completing half of the test trials, whose data were included in behavioral analyses but not in computational modeling. Six neurotypical children were not included for further analyses

because of either not understanding the instruction, inattention to the task, or the experiment program crash. Thus, the final sample consisted of 32 autistic children and 41 neurotypical children, matched for age and IQ.

Supplementary Methods 2: Mini meta-analysis and power analysis

Since no objective justification of the smallest effect size of interest was available for the autism research on cognitive functions, we relied on the subjective measure of that, which was the expected effect size from the related literature[1,2]. To obtain the mean effect size, we conducted a mini meta-analysis of 57 effect sizes from 23 studies to estimate a typical magnitude of the effects of interest. These included learning and decision-making studies that were broadly related to the theoretical background of the current study and more directly related to information sampling and exploration studies. Given the heterogeneous nature of examined studies of autistic traits or diagnosis, we used absolute effect sizes to estimate the typical magnitude of associations or differences in the literature. We took this approach because: 1. The meaning of directional effects was not consistent between the studies using different paradigms across domains; 2. Our goal here was to determine typical effect detectability for sample size planning rather than estimate a specific population parameter.
For these reasons, we used a three-level meta-analytic model. Before entering the model, all the effect sizes (e.g., η^2 and Cohen's *d*) were first converted to correlation *r* and then transformed into Fisher z. The pooled correlation based on the model was $r = .406$ (95% CI [.324, .482]). The estimated variance components were $\tau^2_{\text{Level 3}} = 0.040$ and $\tau^2_{\text{Level 2}} = 0.002$, which means that $I^2_{\text{Level 3}} = 71.12\%$ of the total variance can be attributed to between-study as we expected, and $I^2_{\text{Level 2}} = 3.92\%$ to within-study heterogeneity.

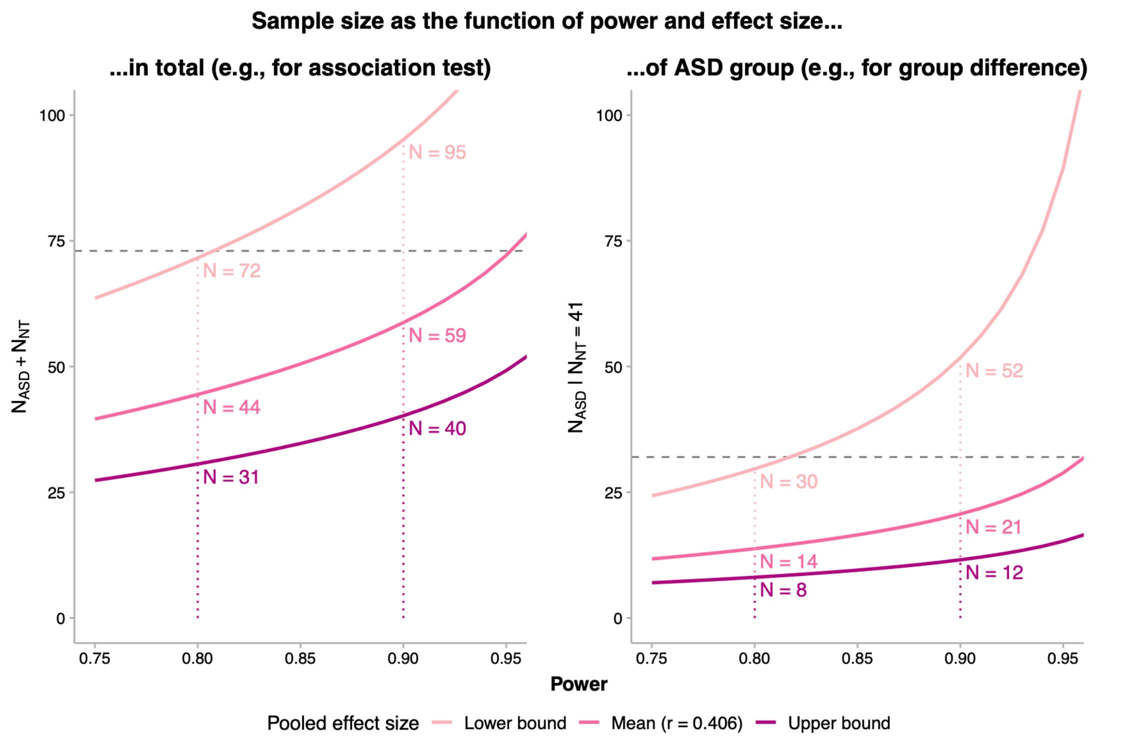

Supplementary Figure 1. A-priori power analysis on sample sizes given expected effect sizes, desired power, and significance level.

Based on the effect size estimates, we did an a-priori power analysis on required sample sizes and powers to detect the effect of interest (Supplementary Figure 1). For the mean pooled effect size (*r* = .406) and significance level = .05, it would need 44 (or 59) participants of two groups in total to reach 80% (or 90%) power, or 14 (or 21) participants in the autistic group given our study design (i.e., group difference against the neurotypical group of 41 children). Given the heterogeneity and bias in the literature, we also considered a more conservative approach to use the lower bound of the confidence interval of pooled effect size (c.f. [3]). With the lower bound effect size (*r* = .324), a total of 72 (or 95) participants of two groups, or 30 (or 52) participants in the autistic group are required to give 80% and 90% power.

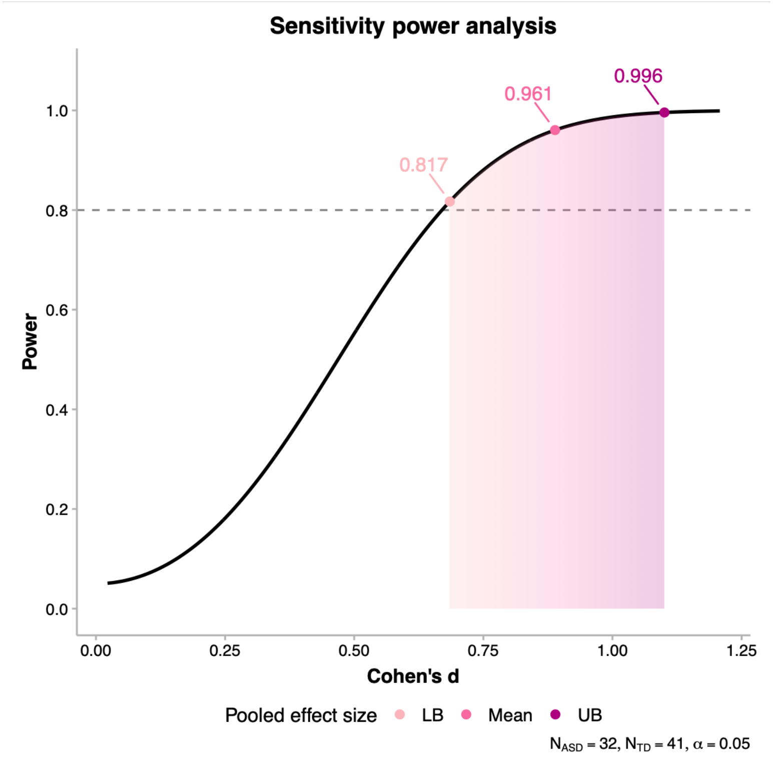

Supplementary Figure 2. Sensitivity power analysis given expected effect sizes and our study design.

Since our sample size, particularly of the autistic children group, was also under resource constraints, we also did a sensitivity power analysis to examine which power we would have for the a-priori ranges of effect sizes of interest (i.e., not for post hoc effect sizes from our study). For our current study design, with 32 autistic and 41 neurotypical children and significant level of .05, we could have approximately 82% and 96% power to detect a group difference with Cohen's d = 0.69 (i.e., the lower bound estimate converted from correlation r) and 0.89 (the mean estimate), respectively.

Supplementary Methods 3: Task instructions and comprehension check questions

Note that the task instructions and the comprehension questions were administered in the local language at each experiment site.

Task instructions

"Today, you're a little explorer who will go on an adventure to either Doggy Island or Kitty Island. Look, there are more doggies on Doggy Island and more kitties on Kitty Island. Your task is to figure out whether you're on Doggy Island or Kitty Island. On the island, you'll meet some small animals. Look here, you've encountered two cats and one dog. These three animals might come from Doggy Island or Kitty Island. If you think there are more kitties than doggies now, where do you think we are more likely to be – on Kitty Island or Doggy Island? Since there are more kitties on Kitty Island, it's more likely that we'll meet more kitties there. Besides adventuring in winter, we'll also go on adventures in summer. Look, during summer, the difference in the number of doggies and kitties is even smaller, making it harder to guess. Look at the top of this screen, there are 100 cookies to feed the kitties and doggies we meet. We might meet three types of animals. First, there are the tiny animals. They are so small that we don't need to feed them cookies. No matter how many tiny animals we meet, the number of cookies won't decrease. We may also meet animals of regular size. For each regular-sized animal we meet, they will eat one cookie, like now, we've met one and there's one less cookie. The more of these animals we meet, the fewer cookies we'll have left. Finally, we might meet big animals. Big animals are so large that they eat four cookies each time we meet one. So, the more big animals we meet, the quicker our cookies will disappear. You will only get coins as a reward if you successfully determine whether you're on Doggy Island or Kitty Island. Sometimes, you might not guess correctly, and then you won't get any coins. But that's okay because unexpected things can happen. Just try to make as many successful guesses as you can. The more coins you get, the more sticker rewards you'll receive. So, in the game, you need to think and decide when you're confident enough to guess where you are based on how many animals you meet, and always pay attention to how your cookies change."

Comprehension check questions.

1. "When you have encountered these three animals, at which island do you think you are more likely to meet them?" (Children were asked for each of the following situations, thus four questions in total.)

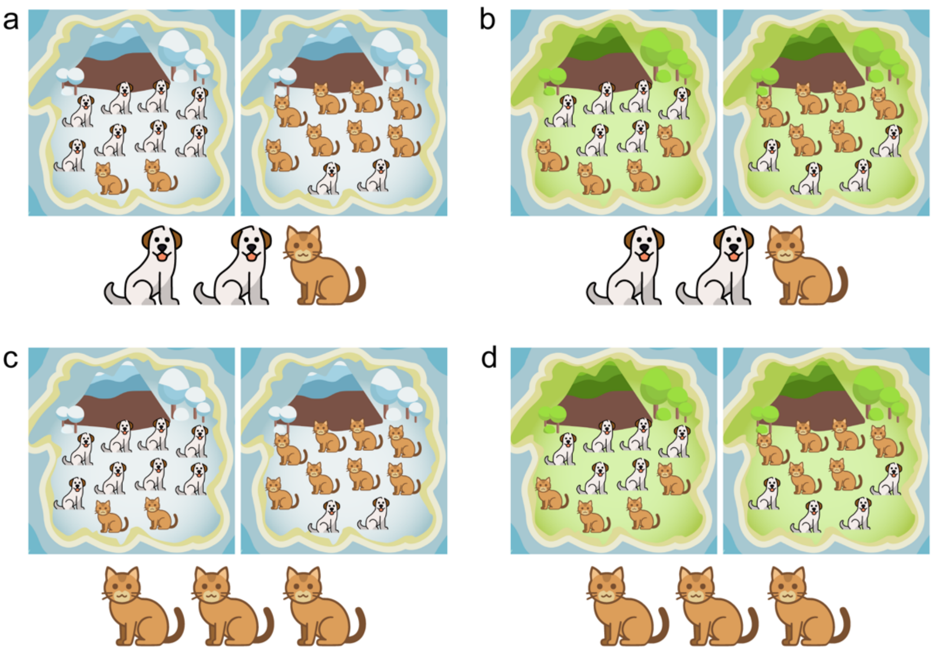

2. “How many cookies does one tiny/regular/big animal eat? If you meet many tiny/regular/big animals, what will happen to your cookies? Will they decrease?” (Children were asked about each type of animals, thus three questions in total.)

Supplementary Methods 4: Parameter specifications of hierarchical Bayesian models

We assume that a generic individual-level parameter, say β, was drawn from a group-level normal distribution; that is, $\beta \sim \text{Normal}(\mu_\beta, \sigma_\beta)$, with μ_β and σ_β being the group-level mean and standard deviation, respectively. Both these group-level parameters were specified with generic weakly informative priors, following the recommendation of Stan Dev team [4]: $\mu_\beta \sim \text{Normal}(0, 1)$ and $\sigma_\beta \sim \text{Half-t}(4, 0, 0.5)$, except for α_{Decay}, which is narrower $\sigma_\alpha \sim \text{Half-t}(4, 0, 0.1)$ given that it will go through inverse logit transform. Since the number of groups was small, particularly in our autistic sample, the data might not provide much information on the group-level variance, so we chose a relatively stronger prior information on the scale parameter (i.e., the standard deviation) that allowed more pooling. Several parameters were constrained within [0, 1] (with inverse logit transform), including decay parameter α_{Decay} in models with decayed evidence and second-thought probabilities.

Supplementary Methods 5: Simulation-based calibration

Considering the possibility that unbalanced data of the autistic group (i.e., six children only completed the 48-trial version of the task) might bias the estimation of group-level parameters, we conducted our simulation-based calibration specifically for the autistic group: when simulating the behavioral data, we used the actual 48-trial stimulus sequences from these six children mixed with the regular 96-trial sequences from other children, fully mimicking the model fitting procedure for the autistic group. This provides a realistic test of whether our hierarchical model can reliably cover parameters even when data includes a mix of trial numbers.

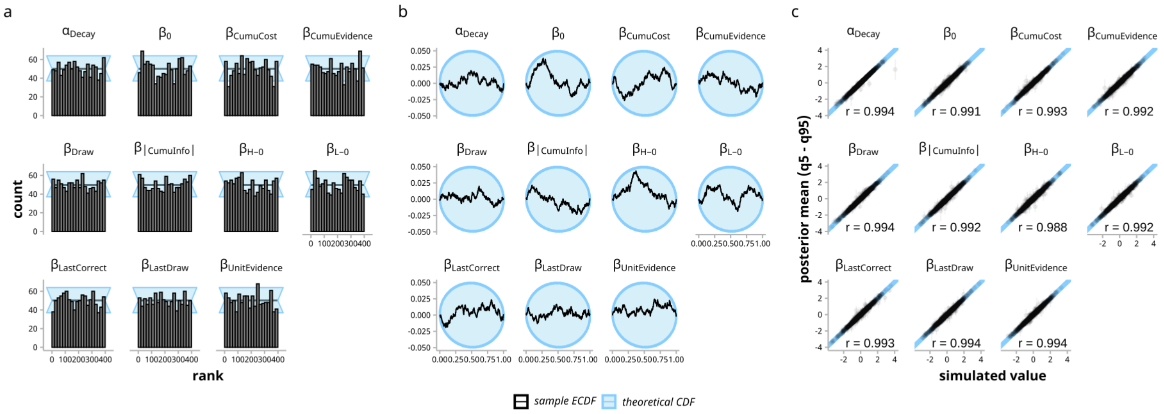

Supplementary Figure 3. Simulation-based calibration results. (a) Distributions of rank statistics of group-level parameters from simulation-based calibration analysis. All these distributions look uniform, supporting that our model is well-calibrated. Light blue ribbons represent approximate 95% confidence intervals; however, these are not very reliable since they are derived by assuming the bins are independent (which they are not). (b) To remedy this, one could look at the empirical cumulative density function (ECDF) plot, which shows the difference between a perfectly uniform distribution and the ECDF. Blue ellipses represent expected deviations at the 95% confidence level. This again demonstrates that our model is well-calibrated. (c) Correlation plots showing the relationship between simulated parameter values and posterior means of recovered parameter distributions.

Supplementary Table 1: Studies and effect sizes included in the mini-meta-analysis

Domain	Authors	Year	*r*	*N*
Information sampling & exploration	Brosnan et al.	2014	0.78	60
			0.83	60
	Farmer et al.	2021	-0.3464102	70
			-0.3	70
			-0.2828427	70
	Jansch & Hare	2014	-0.5176438	43
	Krol & Krol	2019	0.46	44
			0.47	44
	Lu et al.	2019	0.2536	104
			0.2221847	104
			0.2466847	104
	Poli et al.	2024	0.41	70
			0.32	70
	Sahuquillo-Leal	2019	0.3447516	86
	Vella et al.	2018	0.3629984	67
	Young et al.	2016	-0.5	35
Learning & decision-making	Crawley et al.	2020	0.3	145
			0.1872392	204
			0.4581635	204
			0.2135744	223
			0.1467941	223
			0.1385855	223
	De Martino et al.	2008	0.44	29
			0.41	27
			0.46	29
	Goris et al.	2021	0.2	165
			0.24	165
			0.13	165
	Lawson et al.	2017	0.44	49
			0.42	24
			0.42	49
			0.33	49
	Manning et al.	2016	0.02208092	85
			0.07305625	85
	Robic et al.	2015	0.53	29
			0.53	29
			0.47	29

Domain	Authors	Year	*r*	*N*
	Sapey-Triomphe et al.	2021	0.4004245	54
			0.52	54
			0.4791969	54
	Sevgi et al.	2019	0.39	36
			0.52	36
	Shah et al.	2016	0.43	40
Perceptual decision-making	Karvelis et al.	2018	0.327	83
			0.175	83
			0.238	83
			0.185	83
	Lawson et al.	2018	0.44	28
			0.33	28
			0.3	28
	Tortelli et al.	2021	0.45	53
			0.51	53
	Turi et al.	2018	0.7	50
	van Boxtel & Lu	2013	0.43	25
			0.31	25
			0.45	30
			0.38	30

Note: *N* = Total sample size. Original reported effect sizes were extracted and those other than correlation coefficients were converted into *r*.

Supplementary Table 2: Model descriptions

Model	One- or two-stage?	Cost-first or evidence-first?	Second-thought probability	Cost-related decision variables	Evidence-related decision variables	N individual-level parameters
Cost only	One	N/A	N/A	A constant, unit cost (three levels with treatment coding), the number of beads sampled, total sampling cost	NA	5
Cost + Evidence	One	N/A	N/A	As above	Unit log evidence, absolute value of **decayed** cumulative information, total **decayed** log evidence, last trial sample numbers, last trial correctness	11
Cost $\xrightarrow[\text{C-cond}]{\text{continue}}$ Evidence	Two	Cost-first	Controlled by cost conditions (three parameters)	As above	As above, plus a constant	15
Cost $\xrightarrow[\text{E-cond}]{\text{continue}}$ Evidence	Two	Cost-first	By evidence conditions (two parameters)	As above	As above	14
Evidence $\xrightarrow[\text{C-cond}]{\text{continue}}$ Cost	Two	Evidence-first	By cost conditions	As above	As above	15
Evidence $\xrightarrow[\text{E-cond}]{\text{continue}}$ Cost	Two	Evidence-first	By evidence conditions	As above	As above	14

Supplementary Table 3: Group Statistics of Behavioral Measures

	**Credit total**		**Credit in correct trials**		**Accuracy**		**Efficiency**		**Sample bias**		**Sample variation**	
**Conditions**	ASD	NT	ASD	NT	ASD	NT	ASD	NT	ASD	NT	ASD	NT
Ratio: 60%:40%												
Zero: 0	76.54	72.41	100.00	100.00	1.23	0.98	0.95	0.91	-3.10	-7.58	3.61	3.75
	(2.73)	(2.34)	(0.30)	(0.26)	(0.13)	(0.10)	(0.01)	(0.01)	(0.87)	(0.76)	(0.36)	(0.31)
Low: 1	65.11	66.05	89.88	92.02	0.99	0.96	0.96	0.96	-3.04	-5.13	3.94	2.58
	(1.97)	(1.70)	(0.84)	(0.74)	(0.12)	(0.10)	(0.01)	(0.00)	(0.81)	(0.72)	(0.32)	(0.28)
High: 4	46.16	53.92	68.90	77.88	0.78	0.83	0.82	0.91	6.67	4.40	2.91	1.84
	(2.40)	(2.09)	(2.40)	(2.12)	(0.12)	(0.11)	(0.02)	(0.02)	(0.58)	(0.52)	(0.27)	(0.24)
Ratio: 80%:20%												
Zero: 0	91.67	94.05	100.00	100.00	2.73	2.95	0.97	0.95	-3.78	-8.48	3.14	2.60
	(1.79)	(1.52)	(0.27)	(0.22)	(0.24)	(0.21)	(0.01)	(0.01)	(0.99)	(0.87)	(0.36)	(0.32)
Low: 1	78.59	87.78	90.05	93.89	2.18	2.83	0.93	0.96	2.68	-0.96	3.96	2.26
	(1.84)	(1.59)	(0.72)	(0.63)	(0.20)	(0.20)	(0.01)	(0.01)	(0.70)	(0.62)	(0.33)	(0.29)
High: 4	61.86	75.40	72.58	81.78	2.19	2.67	0.83	0.92	3.72	1.47	2.48	1.66
	(2.23)	(1.91)	(2.29)	(2.01)	(0.21)	(0.20)	(0.02)	(0.02)	(0.56)	(0.49)	(0.27)	(0.23)

Note: Mean (SD): estimated marginal means (EMM) and standard errors (SE) from linear mixed models.

Supplementary Table 4: Summary statistics of group-level mean parameters of the best-fitting model

		Group-level mean		
	Parameter	Autistic group[1]	Neurotypical group[1]	Difference[1]
(Constant: intercept)	Zero-cost	-3.75 [-4.31, -3.14]	-2.86 [-3.42, -2.27]	-0.9 [-1.72, -0.07]
Cost-related	Sample numbers	0.74 [0.25, 1.19]	1.7 [1.07, 2.32]	-0.96 [-1.73, -0.19]
	Low- to zero-cost	1.13 [0.66, 1.59]	0.31 [-0.02, 0.65]	0.82 [0.26, 1.4]
	High- to zero-cost	0.75 [0.27, 1.24]	-0.05 [-0.53, 0.44]	0.8 [0.12, 1.5]
	Cumulative cost	0.84 [0.61, 1.08]	1.34 [0.89, 1.78]	-0.5 [-1.02, -0.01]
Evidence-related	Unit log evidence	0.11 [-0.06, 0.28]	0.27 [0.15, 0.39]	-0.16 [-0.37, 0.04]
	Last trial correctness	0.04 [-0.02, 0.11]	0.14 [0.07, 0.21]	-0.1 [-0.19, 0]
	Last trial sample numbers	-0.47 [-0.58, -0.36]	-0.98 [-1.21, -0.76]	0.51 [0.26, 0.76]
	Absolute cumulative information	-0.18 [-0.82, 0.46]	0.71 [0.45, 0.97]	-0.89 [-1.59, -0.22]
	Cumulative log evidence	0.06 [-0.1, 0.24]	-0.07 [-0.14, 0]	0.14 [-0.04, 0.32]
	Evidence decay[2]	0.19 [0.02, 0.51]	0.76 [0.46, 0.93]	-0.54 [-0.84, -0.13]

Note: [1]Median [95% HDI]; [2]Inverse logit transformed to [0, 1]

Supplementary References

1. Lakens, D. Sample Size Justification. Preprint at https://psyarxiv.com/9d3yf/ (2021).
2. Lakens, D., Scheel, A. M. & Isager, P. M. Equivalence Testing for Psychological Research: A Tutorial. *Adv. Methods Pract. Psychol. Sci.* **1**, 259–269 (2018).
3. Perugini, M., Gallucci, M. & Costantini, G. Safeguard Power as a Protection Against Imprecise Power Estimates. *Perspect. Psychol. Sci.* **9**, 319–332 (2014).
4. Stan Development Team. Prior Choice Recommendations. *Github* https://github.com/stan-dev/stan/wiki/Prior-Choice-Recommendations (2024).
