# Supplementary material for: Autistic children sample costly information with increased variability due to inflexible updating
Source: Commun Psychol. 2026 Mar 20;4:80. doi: 10.1038/s44271-026-00439-2 (PMC13168704; doi:10.1038/s44271-026-00439-2)
Supplement: Supplementary file 3 — Reporting Summary [file 44271_2026_439_MOESM3_ESM.pdf]

Corresponding author(s): Hang Zhang, Li Yi

Last updated by author(s): Mar 2, 2026

Reporting Summary

Nature Portfolio wishes to improve the reproducibility of the work that we publish. This form provides structure for consistency and transparency in reporting. For further information on Nature Portfolio policies, see our Editorial Policies and the Editorial Policy Checklist.

Statistics

For all statistical analyses, confirm that the following items are present in the figure legend, table legend, main text, or Methods section.

n/a	Confirmed	
☐	☒	The exact sample size (*n*) for each experimental group/condition, given as a discrete number and unit of measurement
☐	☒	A statement on whether measurements were taken from distinct samples or whether the same sample was measured repeatedly
☐	☒	The statistical test(s) used AND whether they are one- or two-sided<br>*Only common tests should be described solely by name; describe more complex techniques in the Methods section.*
☐	☒	A description of all covariates tested
☐	☒	A description of any assumptions or corrections, such as tests of normality and adjustment for multiple comparisons
☐	☒	A full description of the statistical parameters including central tendency (e.g. means) or other basic estimates (e.g. regression coefficient) AND variation (e.g. standard deviation) or associated estimates of uncertainty (e.g. confidence intervals)
☐	☒	For null hypothesis testing, the test statistic (e.g. *F*, *t*, *r*) with confidence intervals, effect sizes, degrees of freedom and *P* value noted<br>*Give P values as exact values whenever suitable.*
☐	☒	For Bayesian analysis, information on the choice of priors and Markov chain Monte Carlo settings
☐	☒	For hierarchical and complex designs, identification of the appropriate level for tests and full reporting of outcomes
☐	☒	Estimates of effect sizes (e.g. Cohen's *d*, Pearson's *r*), indicating how they were calculated

Our web collection on statistics for biologists contains articles on many of the points above.

Software and code

Policy information about availability of computer code

Data collection	Data were collected in MATLAB 2016b (The MathWorks inc.) with Psychtoolbox-3.0.14 package.
Data analysis	Custom scripts for tidying raw data and statistical modeling with packages in R v4.3.2. R packages include: tidyverse_2.0.0, afex_v1.2-1, emmeans_1.8.5, cmdstanr_0.5.3, loo_2.6.0. cmdstanr use cmdstan engine for Bayesian estimation (v2.32.0).

For manuscripts utilizing custom algorithms or software that are central to the research but not yet described in published literature, software must be made available to editors and reviewers. We strongly encourage code deposition in a community repository (e.g. GitHub). See the Nature Portfolio guidelines for submitting code & software for further information.

Data

Policy information about availability of data

All manuscripts must include a data availability statement. This statement should provide the following information, where applicable:

- Accession codes, unique identifiers, or web links for publicly available datasets
- A description of any restrictions on data availability
- For clinical datasets or third party data, please ensure that the statement adheres to our policy

All the behavior data supporting the analyses and conclusions of the article are available at https://doi.org/10.17605/OSF.IO/WDTQ2

Human research participants

Policy information about studies involving human research participants and Sex and Gender in Research.

Reporting on sex and gender	We used the term "gender" to refer to either parental reports or children's self-reports of their gender. While our samples included both boys and girls, autism spectrum disorder has a significantly higher prevalence in boys. To match the gender ratio in the autistic child sample, the majority of neurotypical children in our study were also boys. We provided the numbers and ratios of children of different genders in both the autistic and neurotypical child samples.
Population characteristics	See below.
Recruitment	We recruited children from schools (including normal kindergartens and special education schools) and posters for the local community. The parents were first notified by the school teachers about the experiments and then signed up for our testings. We then recruited children from the pool who met the criteria (e.g., age, IQ, diagnosis). The parents whose children have more severe behavioral or academic problems might be more inclined to sign up for our studies since we also included multiple standard psychological testings in our series of studies, particularly the IQ testing. However, since we had requirements for age and IQ levels, only children aged from 5 to 8 with normal intellectual ability were recruited with age and IQ matched between autistic and neurotypical children. Thus, the observed differences in autistic children may only apply to verbal autistic children. Controlling for ages and IQ scores did not change our results.
Ethics oversight	Committee for Protecting Human and Animal Subjects, School of Psychological and Cognitive Sciences, Peking University

Note that full information on the approval of the study protocol must also be provided in the manuscript.

Field-specific reporting

Please select the one below that is the best fit for your research. If you are not sure, read the appropriate sections before making your selection.

☐ Life sciences ☒ Behavioural & social sciences ☐ Ecological, evolutionary & environmental sciences

For a reference copy of the document with all sections, see nature.com/documents/nr-reporting-summary-flat.pdf

Behavioural & social sciences study design

All studies must disclose on these points even when the disclosure is negative.

Study description	The study analyzed quantitative behavioral data from an information sampling task. The experiment employed a mixed design, with autism diagnosis as the between-group factor and information costs and gains as two within-group factors. Additionally, we collected neuropsychological measures from the children.
Research sample	The data analyses included 32 autistic children (aged 5.11–8.43 years, mean = 6.4; 6 females; mean full-scale IQ = 105.4) and 41 neurotypical children (aged 5.02–7.68 years, mean = 6.45; 11 females; mean full-scale IQ = 111.4), matched on gender, age, and IQ. The age ranges were selected to focus on early childhood, a developmental period of interest, while the IQ ranges ensured participants could successfully complete the cognitive tasks designed to assess decision-making behaviors. Given that many autistic children have intellectual disabilities and are non-verbal, our samples were neither intended nor able to be representative of the broader autistic population.
Sampling strategy	Our sampling strategy combined convenience sampling (parents were contacted and signed up voluntarily), purposive sampling (we selected children from those whose parents signed up and met predetermined criteria for age and IQ), and quota sampling (we recruited neurotypical children to match the age and IQ of the verbal autistic group). The sample size was determined by an a priori power analysis based on a mini meta-analysis of 23 related studies (r = .406), which indicated that our final sample size provided >80% power to detect the effects of interest.
Data collection	Data were collected using a laptop connected to an external 24-inch monitor for stimulus presentation and a game controller for input. Full-scale IQ was assessed using the Chinese version of the Wechsler scales, administered by members of the research team. Scores were recorded on paper and later converted using an online scoring service. Parents of participating children were present alongside the children and researchers during the sessions. Due to the study design and recruitment procedures, blinding was not feasible. The experiment was conducted on-site at different schools where groups of children were recruited. Using a within-group design, all children were exposed to the same conditions, with information presented on the screen.
Timing	Data collection started from June 2019 to March 2023, and March to July 2025
Data exclusions	Six autistic children did not understand the instructions to pass the practice trials, and thus they did not take test trials. Six neurotypical children were not included for further analyses because of either not understanding the instruction (i.e., failed to pass the practice trials), evident inattention to the task, or the experiment program crash. Exclusion criteria were pre-established.

Non-participation	One autistic child quit the experiment during the break after finishing the first half of the task due to emotional distress.
Randomization	Since the between-group factor (i.e., diagnosis) was observational, there was no randomization for that. For the two within-group factors, the experiment contained three blocks each for one of three information cost conditions, with the order counterbalanced across participants. Each block nested two mini-blocks with different evidence conditions in a random order.

Reporting for specific materials, systems and methods

We require information from authors about some types of materials, experimental systems and methods used in many studies. Here, indicate whether each material, system or method listed is relevant to your study. If you are not sure if a list item applies to your research, read the appropriate section before selecting a response.

Materials & experimental systems

n/a	Involved in the study
☒	☐ Antibodies
☒	☐ Eukaryotic cell lines
☒	☐ Palaeontology and archaeology
☒	☐ Animals and other organisms
☒	☐ Clinical data
☒	☐ Dual use research of concern

Methods

n/a	Involved in the study
☒	☐ ChIP-seq
☒	☐ Flow cytometry
☒	☐ MRI-based neuroimaging
